# Effects of Socialization on Problem Solving in Domestic Cats

**DOI:** 10.3390/ani14172604

**Published:** 2024-09-07

**Authors:** Preston Foerder, Mary C. Howard

**Affiliations:** 1 Psychology Department, CHASS, University of Detroit Mercy, Detroit, MI 48221, USA; 2 Psychology Department, University of Tennessee at Chattanooga, Chattanooga, TN 37403, USA; marycatehoward4@gmail.com

**Keywords:** *Felis catus*, sociality, puzzle box, cognition, age effects

## Abstract

**Simple Summary:**

Research has shown that animals that are used to interacting with human beings are better at problem solving. We socialize domestic cats by taking advantage of a sensitive period in their development to make them more amenable to humans so that we can keep them as pets. We studied if this socialization makes the cats better able to solve problems by giving them a puzzle box with a food reward. We studied cats from an animal shelter that used tests to determine incoming cats’ socialization. We found that more socialized cats were more likely to solve the puzzle and solve it faster. We can use this information to tell us about cat socialization, cat cognition, and the effects of human exposure on other animals.

**Abstract:**

Domestic cats are capable of leading both solitary and social lives and socializing to humans. This type of socialization may also enhance an animal’s problem-solving ability. We examined the relationship between socialization and problem-solving ability, problem-solving speed, and latency to approach a novel apparatus in domestic cats. Socialization towards humans was measured with the Feline Behavior Assessment based on the ASPCA’s Feline Spectrum Assessment. This modified measure requires assessors to observe an individual cat’s behavior during three steps: observation test, door test, and the stroke and push test. During each test, the assessor examined specific behaviors that are indicative of socialization. Problem solving was assessed with a food-acquisition puzzle box that required the subject to pull on a tab to release a food reward. Twenty-four out of eighty-six cats solved the problem-solving task. More socialized cats were more likely to solve the problem, solve it faster, and approach the apparatus sooner. We also found a significant relationship between age and problem solving; younger adult cats were more likely to solve the problem than older adults. These results provide evidence that domestic cats are not only capable of solving this type of problem but also that their socialization towards humans influences their abilities.

## 1. Introduction

Domestic cats (*Felis catus*) are extremely adaptable animals that can function and survive in many different environments. Extensive learning abilities allow these animals to adapt rapidly to new environments [1]. While research on cat cognition is sparse compared to domestic dogs, there has been research that focuses on their cognitive abilities, such as maze performance, trial-and-error learning, and observational learning [2,3,4,5]. Although cats were used as subjects in Thorndike’s [5] landmark learning research, since then, the species has fallen out of favor for such research. Thorndike studied the cats as they solved the problem of food acquisition from within a puzzle box. Such problem-solving tasks have become a primary method of measuring animals’ learning, cognitive, and innovative abilities [6,7]. Problem solving provides information about behavioral flexibility [8]. Encountering a novel problem, such as obtaining food that is out of reach, and being able to adjust to the environment are essential for survival in the wild [6].

Two factors that can affect animal cognition are their sociality and socialization [9,10,11]. Sociality refers to the relationships formed by individuals cohabiting and interacting with one another [12]. Research on the influence sociality has on cognitive ability is typically focused on providing evidence for the social intelligence hypothesis [13], which posits that intelligence evolved due to the challenges of dealing with complex social relationships formed between animals [13]. Socialization is defined as a process that determines an animal’s comfort level or social character towards others, including humans [14,15]. Socialization has also been studied in regard to the effects of habituation to humans on captive animal behavior [10,11]. In domestic cats, socialization happens during a “sensitive period” between two and seven weeks of age. At this time, cats socialize with their mothers. We use this period to form a social attachment between cats and humans to facilitate their role as house pets [14].

Captivity effects that habituate animals to humans seem to affect performance on cognitive tests. Research on two species of orangutans (*Pongo abelii* and *Pongo pygmaeus*) investigated the effect of captivity and orientation towards humans, defined as an animal’s reaction towards an unfamiliar human, on a problem-solving task. Greater human orientation, brought about by increased human contact, affected the animals’ motivation to explore, which affected their ability to solve the problem [10]. Further research found that curiosity, indicated by less neophobia, was instilled by captivity [11]. Similar effects were found in vervet monkeys (*Chlorocebus pygerythrus*) [16]. Likewise, captive spotted hyenas (*Crocuta crocuta*) show greater innovative problem-solving abilities than wild hyenas. This facility may also be due to the human interaction derived from captivity [17].

The domestic cat is able to form unique interspecific social relationships with humans [1]. However, cats can vary tremendously in how well they are socialized to humans, ranging from feral to lap cats [1]. Interspecific relationships typically only occur if kittens are exposed to another species during their sensitive period in early life [2]. Since attempting to socialize a cat outside of its sensitive period can be quite challenging, animal shelters have found that it is important to identify or measure a cat’s socialization towards humans to provide the best care for them in their adoptive homes [15].

The range of socialization in cats and the interest of animal shelters in determining levels of socialization provide a unique opportunity to study the relationship between socialization towards humans and problem-solving ability across a fairly large sample. We investigated the relationship between socialization and problem-solving skills in domestic cats. Our subjects were domestic cats from the McKamey Animal Center (MAC) in Chattanooga, TN, USA. Many of the cats were assigned socialization grades with MAC’s Feline Behavior Assessment, based on aspects of the American Society for the Prevention of Cruelty to Animals’ (ASPCA) Feline Spectrum Assessment [18]. We administered a problem-solving task, which involved obtaining food from a puzzle box, to each cat individually to investigate the relationship between their problem-solving abilities and their socialization scores. We hypothesized that (1) more socialized cats would be more likely to solve the problem-solving task than less socialized cats; (2) more socialized cats would approach the apparatus sooner than less socialized cats; and (3) more socialized cats would complete the task more quickly than the less socialized cats. We also examined a number of other variables for their effects on problem-solving: age, sex, spaying/neutering, and time spent at the shelter.

## 2. Materials and Methods

### 2.1. Subjects and Housing

Our subjects were 86 cats from MAC in Chattanooga, TN, USA, 40 m, 46 f. Ages ranged from 1 to 10 years (*M* =3.472, *SD* = 2.29) and number of days at the shelter ranged from 8.70 to 153.0 (*M* = 44.23, *SD* = 16.89). All cats in the study were singly housed. Fifty-one of the cats, 25 m, 26 f, that were tested on the constructed apparatus were previously assessed at MAC’s discretion using their Feline Behavior Assessment, which noted how many socialized behaviors the cats displayed during an observational four-step test. All cats were vaccinated and checked for health issues prior to participation. Kittens and females in heat were excluded due to a lack of consistent socialized behaviors [19]. Cats were tested as they became available. There were no other preconditions as to which cats were used in the study.

All research took place at MAC which is a nonprofit animal facility with a municipal contract with the city of Chattanooga, TN. Their staff consists of paid employees and volunteers. All cats are individually housed. Testing took place in the cat’s home cage, measuring 60.96 cm W × 50.8 cm D × 76.2 cm H. The cages were arranged against a wall, 3 high and 6 across. Each cage had an openable steel wire grid door.

### 2.2. Materials

**Figure 1:**
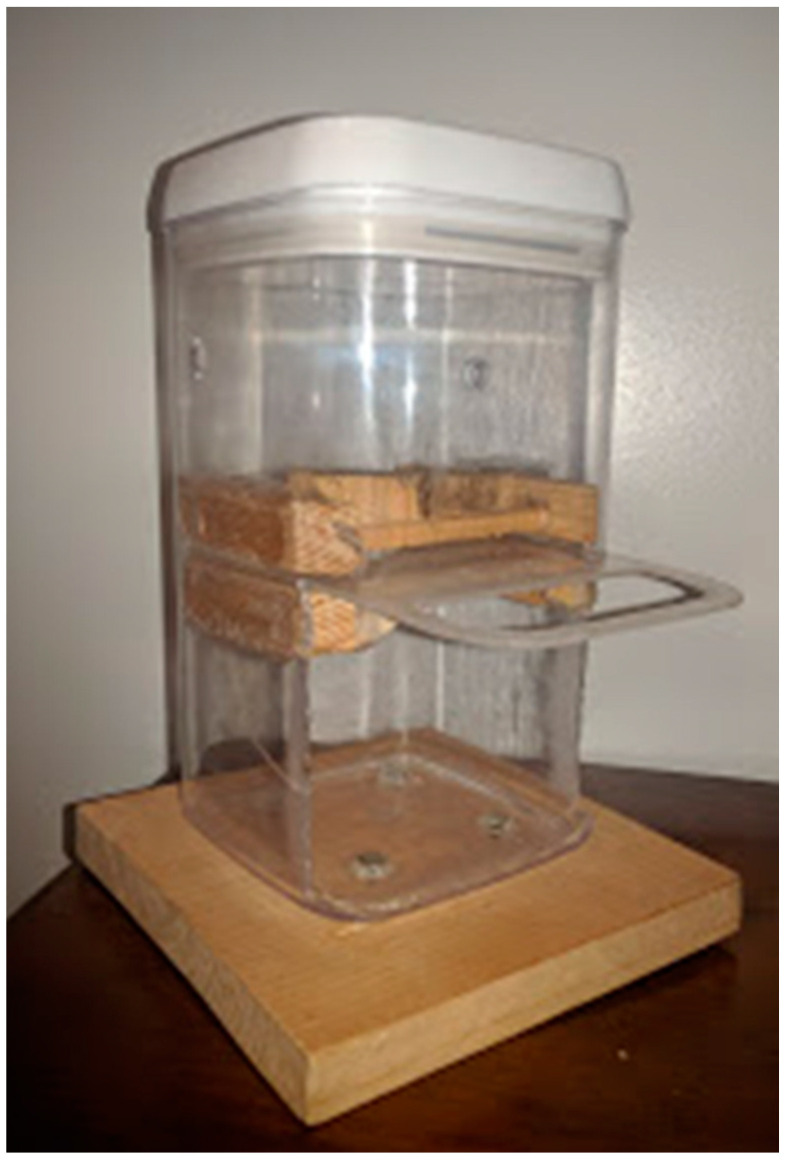
Problem-solving puzzle box.

Problem-solving ability was measured using a puzzle box adapted from Thornton and Samson [7] (see Figure 1). The food reward was Orijen Six Fish Treats^®^, which was chosen from a preference test. The puzzle box was a transparent, plastic container measuring 12.06 cm W × 12.06 cm D × 22.86 cm H with perforations so that the cat could see and smell the food reward that was placed inside. The apparatus was mounted on a wooden base measuring 16.51 cm W × 17.14 D × 1.90 cm H. It had two levels that were separated by a sheet of plastic that had a tab that the cats could pull with either their paws or teeth to obtain the treat. The treat was placed in the top level sitting on top of the sheet of plastic. The bottom level of the apparatus had an opening for the cat to obtain the reward when it fell. To successfully obtain the food reward, the cats had to pull the tab separating the two levels, which allowed the treat to fall into the cat’s reach.

MAC tested socialization using the Feline Behavior Assessment (FBA), which is a modified version of the ASPCA’s Feline Spectrum Assessment (FSA) [18,20], to better fit the center’s requirements (see Supplemental Materials). The ASPCA’s assessment used two slightly different protocols for morning and afternoon assessments, while MAC only used one protocol for all assessments. Also, the FSA has one step using an interactive toy, while the FBA has a step involving a live cat. At the time of our research, the FBA had been in use for 2 years. The FBA is administered at least two hours after intake processing, by at least two different employees, and not immediately before or after feeding times. Cats were FBA-tested based on availability of staff. Not all cats were tested.

All cat interactions with the puzzle box were recorded with an HD Canon Vixia HF R400 (Canon USA. Melville, NY) video recorder attached to a tripod. A DBPower EX5000 camera connected to an iPad 4 MD528LL/A via Wi-Fi allowed for remote viewing of the sessions. Videos were coded using the Behavioral Observational Research Interactive Software v 3.60 (BORIS) [21]. We conducted statistical analyses using IBM SPSS Statistics (version 25, Armonk, NY USA).

### 2.3. Procedure

#### 2.3.1. Feline Behavior Assessment (FBA)

The FBA uses a four-step observational method to note how many socialized behaviors are displayed. Steps one to three are tested while the cat is in their cage and step four occurs outside the cage. Step 1, the observation test, lasts ~30 s. Upon first interaction with the cat, the assessor is instructed to observe the cat’s behaviors when they approach their cage in a non-threatening manner. The goal for this step is to determine the cat’s response to an approaching person. Step 2, the door test, lasts ~30 s. The assessor cracks the cage door open and places their palm in the cage, out of reach of the cat, so that the cat must initiate an interaction. This step’s purpose is to identify how the cat responds to the invitation of human touch. Step 3, the stroke and push test, has no specified time limit. The assessor uses a backscratcher to reach in the cage and holds it in front of the cat’s nose to allow it to be smelled. Next, they slowly move the backscratcher to stroke the cat’s cheek, chin, and back, gently pushing down between the cat’s shoulder blades. This step tests response to a gentle touch and sensitivity to restraint. Step 4, the cat test, requires the assessor to remove the cat from its cage and hold it in front of another cat’s cage for ~15 s to identify if the cat is suitable to live in a home with multiple animals. Step 4 is only tested on cats that respond ambiguously in steps 1–3, and MAC needs further information to assess them properly. As not all cats receive step 4, scores from this step were not used in calculating socialization scores for this study.

The socialized behaviors are broken down into “A” behaviors and “B” behaviors (see Supplemental Materials). “A” behaviors consisted of the following: meows, rubs on bars, kneads, touches bars, at the front of cage, and tail is up. “B” behaviors consisted of the following: yawns, grooms, shakes, approaches front, sniffs, rolls, reaches, and still standing or moving at the end. “A” behaviors were considered to be distance-reducing behaviors and “B” behaviors may be indicative of socialization [22]. “A” behaviors show that the cat is adequately socialized to people to adjust to the stressful environment of a shelter. Cats that display at least four “B” behaviors are considered socialized. Cats that do not display at least four, are not determined to be socialized or could take longer to adjust to a new environment. Cats receive a score of total “A” behaviors from 0 to 54, and “B” behaviors from 0 to 72. A high number in each category indicates that the cat is extremely socialized. To create a single socialization score, the cats’ total “A” and “B” behaviors for all three administrations were weighted together: A + (B × 0.25). “B” behaviors were designated as a fourth of the “A” behaviors in accordance with the FBA scoring.

#### 2.3.2. Inter-Rater Reliability

Videos of the sessions were coded by the researcher and a “blind” undergraduate student coder. BORIS was used to compute a Cohen’s κ to determine inter-rater reliability between the blind rater and researcher.

#### 2.3.3. Problem Solving

All sessions were conducted in between feeding times and after cages had been cleaned, from 1:00 p.m. to 3:00 p.m. Sessions lasted until the cat obtained the food or for a maximum of 10 min. The video camera and DBPower camera were set up outside each cat’s cage to record and monitor the session. During testing, the cat’s food and water dishes were removed to provide more space and ensure the treat was the only source of food during the 10 min session. We attracted the cat’s attention by showing them the treat. When they engaged, we placed the treat inside the apparatus which was placed in the cage. The researcher then left the room for the duration of the session and observed remotely via the iPad which was Wi-Fi-connected to the DBPower camera. MAC staff were in the room during testing but were engaged in cage cleaning away from the testing area and were advised to avoid watching the testing. There was also a radio playing a music station in the room. Sessions ended when the cat solved the puzzle by obtaining and eating the treat or after 10 min. Following testing, the puzzle box was removed, and their food and water were returned to their cage. If the cat either did not solve the task or find the food reward on their own, the treat was given to them. After each session, the apparatus was cleaned with disinfectant wipes commonly used by MAC.

## 3. Results

BORIS was used to compute Cohen’s κ to determine the inter-rater reliability between the blind rater and researcher. We found acceptable agreement between raters (κ = 0.82).

Two cats were excluded from analysis in time 3: one did not have a third FBA score and the other was an outlier, *z* = 3.42. A total of 24 out of 86 cats solved the problem. We conducted a logistic regression analysis to predict the probability that a cat would solve the problem. The predictor variables were age, sex, spay/neuter status, and days previously spent at the center. A test of the full model versus a model with only the intercept was statistically significant, χ^2^(4, *N* = 86) = 16.457, *p* = 0.002. Table 1 shows the logistic regression coefficient, Wald test, odds ratio, and intercept for each of the predictors. At α = 0.05, only age had a significant partial effect, although sex had a nearly significant partial effect.

We conducted an additional logistic regression analysis, adding socialization to the model. A test of the full model versus a model with intercept only was statistically significant, χ^2^(5, *N* = 51) = 25.962, *p* < 0.001. Table 2 shows the logistic regression coefficient, Wald test, odds ratio, and intercept for each of the predictors. At α = 0.05, socialization and age had significant partial effects.

Univariate analysis confirmed the significance of the effect of socialization on problem solving as cats with higher socialization scores (*M* = 24.92, *SD* = 9.85) were more likely to obtain the food than cats with lower socialization scores (*M* = 18.60, *SD* = 10.91); *t*(49) = 2.072, *p* = 0.022, one-tailed. Cats that solved the problem were, on average, younger (*M* = 2.23, *SD* = 1.75) than cats who did not (*M* = 3.95, *SD* = 2.30), *t*(84) = −3.297, *p* = 0.001. No significant correlation was found between socialization and age, *r*(49) = −0.109, *p* = 0.446. No significant effect was found for sex on solving, *t*(84) = −1.839, *p* = 0.070. No difference was found in socialization between sexes, *t*(49) = −0.315, *p* = 0.754.

**Figure 2:**
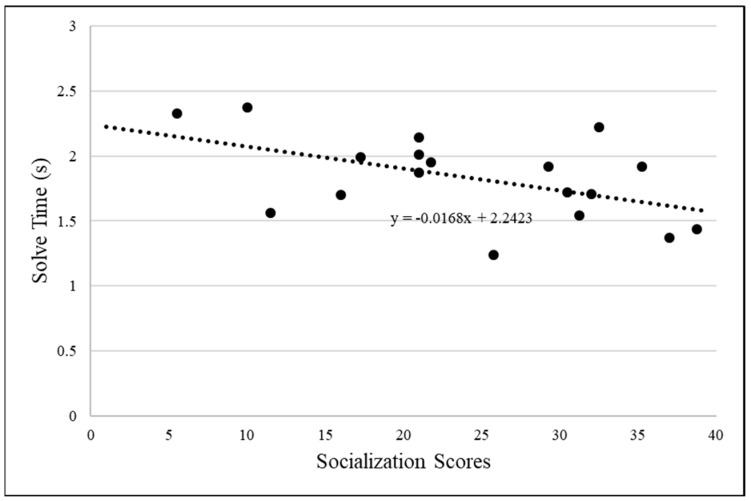
Graph of socialization score with log of solve time.

To assess the relationship between socialization scores and solve times, the time between placement of the apparatus and food acquisition, the logarithm of solve time was computed to ensure that it was symmetric. There was one outlier for solve time, *z* = 3.08, that was excluded from the analysis. The average time the cats solved the apparatus was 83 s (*M* = 82.45, *SD* = 58.11) after the apparatus was placed inside of their cage. We conducted a correlation analysis between socialization scores and solve times (*N* = 19). There was a significant negative correlation *r*(16) = −0.506, *p* = 0.032, higher socialization scores had lower solve times (see Figure 2).

**Figure 3:**
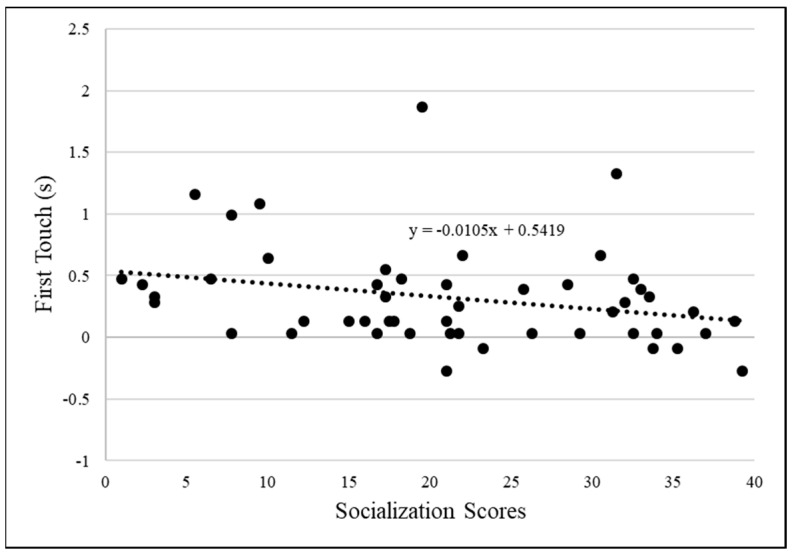
Graph of socialization score with the log of first touch time.

To assess the relationship between socialization scores and time of first touch, scores were converted to logs. There was one outlier for first touch time, *z* = 8.70, that was excluded from the analysis. The time the cats first touched the apparatus after it was placed inside of their cage was *M* = 3.96 s, *SD* = 8.69. There was a negative correlation that trended towards significance between the socialization scores and time of first touch, *r*(48) = −0.28, *p* = 0.052 (see Figure 3).

## 4. Discussion

Supporting our hypotheses, we found that more socialized cats were more likely to solve the food acquisition task than less socialized cats, solve it faster, and (in a near-significant result) touch the puzzle box sooner. This relationship may be because socialization towards humans has a positive influence on their problem-solving abilities. Similarly, Damerius and Forss et al. (2017) found that orangutans who were accustomed to humans through captivity showed greater capability in cognitive testing as opposed to wild orangutans. However, as opposed to wild animals, domestic cats have been purposefully socialized to integrate with humans [14]. There may be aspects of this process that affect their cognitive abilities.

We also found an age effect on problem solving; younger cats were more likely to solve the problem than older cats. We had not anticipated this result. Our cats ranged from young adult to mature adult [23]. Most research on age and problem solving compares juveniles to much older cats. A review of the research in this area has shown very little evidence for an effect of age on problem solving [6]. More recent research on a wide range of wild cat species also showed no effect of age on problem solving [24]. Some research has shown that older animals have an advantage in problem solving. Adult callitrichid monkeys showed more innovation in problem solving than younger ones [25]. We also found no relation between socialization scores and age that may have accounted for this finding. These results are definitely an avenue for future research.

There is a question about what aspect of socialization may lead to problem-solving ability. Certainly, there are aspects of the FBA (and the FSA) that can be seen as testing neophobia, presenting the cats with novel stimuli to see if they are afraid. A lack of neophobia (neophilia) leading to greater curiosity has been indicated in successful problem solving [11,16]. In callitrichid monkeys, neophilia was not found to be indicative of innovation in problem solving [25]. Other research on parrots indicates that the differences in problem solving between wild and captive birds may be a matter of motivation [26]. The unique human bonding aspect of socialization may be responsible for the lack of neophobia that leads to problem solving.

Only about a third of the cats solved the problem (24 out of 86). There could be multiple reasons for this result. Although the problem-solving sessions were conducted with other humans in the room with the cat, they were instructed to avoid observing the testing procedure. It could be that socialization makes the cats more dependent on human cues to solve a problem. However, dependence on humans is unlikely as it has been shown that when faced with an unsolvable problem, unlike dogs, cats do not look to people for help [27]. The cats were only given a single 10 min trial with the puzzle box. A longer trial may have increased the number of cats solving the problem. Also, more experience with the puzzle may have led to increased success in the puzzle through practice effects or reduced neophobia. The cats were not food-deprived and were maintained on their normal feeding schedule. It could have been that the cats were not sufficiently food-motivated. Often, in this type of research, we see a contrafreeloading effect: animals will take food that they have to work for over free food. It has been found that cats, unlike most other tested species, do not show contrafreeloading [28]. This lack of interest in working to get food may also help to explain why only about a third of our cats solved the problem. Future research can be performed to determine if this unique aspect of cat behavior is affecting their problem solving ability. Further research may also be able to determine the differences on cognitive abilities between socialization and mere habituation to humans.

The FBA is an adapted version of the ASPCA’s Feline Spectrum Assessment (FSA) [18]. MAC has removed one of the steps of the FSA, presenting an interactive toy, and added a step, holding up a live cat. The live-cat step was not used in our study because it is not given to all cats tested. FBA also uses a different scoring system than the FSA, which we have quantified for statistical analysis. Even though they had not conducted a research evaluation of the FBA, MAC felt that these changes better suited their needs and that their results were indicative of how socialized their cats were as shown by increased successful adoptions. Our results, correlating their socialization scores with problem solving, lends external validity to their assessment.

We conducted our research in a working animal shelter. There are disadvantages and advantages to this method of conducting research. The disadvantages are a lack of complete control of the testing area, i.e., MAC staff continuing their daily routines. However, this did not seem to affect the cats as indicated by our result that there was no significant effect of amount of time spent at the shelter. An advantage is that we had access to a large sample of cats for the research. We also limited the testing to a single trial for each cat. This procedure may be more amenable to cats as opposed to other species such as dogs that are easier to test over multiple trials. This difference in the animals’ attention levels may account for the higher amount of studies on cognition in dogs as opposed to cats. Indeed, researchers may need to change their procedures to be more amenable to cats’ temperaments to test their cognition [4].

## 5. Conclusions

Our study investigated the effects of socialization on a cat’s problem-solving ability. Given a food acquisition puzzle apparatus, more socialized cats were more likely to solve the problem, solve it faster, and touch the apparatus sooner. We used one animal shelter’s socialization assessment to determine the amount of socialization in each cat. This research aligns with other research showing that wild animals habituated to humans show improved problem-solving abilities. Future research may determine if there are differences between the process of socialization in cats and mere habituation to human beings.

## Figures and Tables

**Table 1:** Logistic regression predicting problem solving from age, sex, spayed/neutered status, and days.

	B	Wald	*p*	Odds Ratio
Age	−0.427	7.664	0.006	0.652
Sex	−1.056	3.791	0.052	0.348
Spay/Neuter	−0.780	0.913	0.339	0.458
Days	0.003	0.166	0.684	1.003
Intercept	1.345	2.036	0.154	3.838

**Table 2:** Logistic regression predicting problem solving from socialization, age, sex, spayed/neuterd status, and days.

	B	Wald	*p*	Odds Ratio
Socialization	0.096	4.134	0.042	1.100
Age	−1.008	9.492	0.002	0.365
Sex	−0.137	0.029	0.866	0.872
Spay/Neuter	−1.384	1.558	0.212	0.251
Days	−0.004	0.126	0.722	0.996
Intercept	1.612	1.009	0.315	5.011

## Data Availability

Data are available upon request.

## Supplementary Materials

The following supporting information can be downloaded at: https://www.mdpi.com/article/10.3390/ani14172604/s1, Table S1: Feline Behavior Assessment score sheet steps 1–4.
