# Supplementary material for: Effects of Socialization on Problem Solving in Domestic Cats
Source: Animals (Basel). 2024 Sep 7;14(17):2604. doi: 10.3390/ani14172604 (PMC11394271; doi:10.3390/ani14172604)
Supplement: Supplementary file 1 [file animals-14-02604-s001.zip › animals-3171068-supplementary.pdf]

Table S1

Feline Behavior Assessment Score Sheet Steps 1-4

(*Feline Behavior Assessment Score Sheet, 2015*)

Cat Temperament Test

ID: __________ Cat's Name: ______________________________ Final Grade: ____________
Dates: ________ Assessors: _______________________________

**Step 1: The Observation Test (30 seconds)** Approach the cage and, speaking in a soft, gentle voice, and slow-blinking, extend your hand			
A	A	A	Chirps
A	A	A	Rubs on bars
A	A	A	Kneads
A	A	A	Touches bars
A	A	A	At the front
A	A	A	Tail is up
B	B	B	Yawns
B	B	B	Grooms
B	B	B	Shakes
B	B	B	Approaches front
B	B	B	Sniffs
B	B	B	Rolls
B	B	B	Reaches
B	B	B	Still standing or moving at the end
**Notes:**			

**Step 2: The Door Test (30 seconds)** Crack the cage door open and observe the cat, then close the door			
A	A	A	Chirps
A	A	A	Rubs on bars
A	A	A	Kneads
A	A	A	Touches bars
A	A	A	At the front
A	A	A	Tail is up
B	B	B	Yawns
B	B	B	Grooms
B	B	B	Shakes
B	B	B	Approaches front
B	B	B	Sniffs
B	B	B	Rolls
B	B	B	Reaches

B	B	B	Still standing or moving at the end
Notes:			

**Step 3: The Stroke and Push Test (No Time Limit)** Reach the backscratcher through the bars and hold in front of the cat's nose to let him sniff. Stroke the cat gently under the chin. Allow him to sniff it again, then stroke again, then gently push down between his shoulder blades.			
A	A	A	Chirps
A	A	A	Rubs on bars
A	A	A	Kneads
A	A	A	Touches bars
A	A	A	At the front
A	A	A	Tail is up
B	B	B	Yawns
B	B	B	Grooms
B	B	B	Shakes
B	B	B	Approaches front
B	B	B	Sniffs
B	B	B	Rolls
B	B	B	Reaches
B	B	B	Still standing or moving at the end
Notes:			

**Step 4: The Cat Test** *Perform ONLY on cats that score in the adoptable rage*. Remove the cat from the cage and hold him up to 3 cats.			
A	A	A	Sniffs, reaches, meows, chirps
B	B	B	No reaction
F	F	F	Hisses/growls
N/A	N/A	N/A	Struggles through entire hold (RETEST)
Cat 1	Cat 2	Cat 3	(Circle one) Ok w/ cats No cats

Time1 Total A's	Time2 Total A's	Time3 Total A's	Overall Total A's
Time1 Total B's	Time2 Total B's	Time3 Total B's	Overall Total B's
